# Gene Expression Analysis Reveals Prognostic Biomarkers of the Tyrosine Metabolism Reprogramming Pathway for Prostate Cancer

**DOI:** 10.1155/2022/5504173

**Published:** 2022-07-06

**Authors:** Wei Li, Zhe Lu, Dongqing Pan, Zejian Zhang, Hua He, Jiacheng Wu, Naixiong Peng

**Affiliations:** ^1^Department of Urology, Shenzhen Longhua District Central Hospital, The Affiliated Central Hospital of Shenzhen Longhua District, Guangdong Medical University, Shenzhen, Guangdong Province, China; ^2^Clinical Laboratory, Women and Children's Health Care Center of Hainan Province, Haikou, Hainan Province, China; ^3^Dachong Health Service Center, Headquarters of Nanshan Medical Group, Shenzhen, Guangdong Province, China; ^4^Department of Urology, Affiliated Tumor Hospital of Nantong University and Nantong Tumor Hospital, Nantong, Jiansu Province, China

## Abstract

**Background:**

Tyrosine metabolism pathway-related genes were related to prostate cancer progression, which may be used as potential prognostic markers.

**Aims:**

To dissect the dysregulation of tyrosine metabolism in prostate cancer and build a prognostic signature based on tyrosine metabolism-related genes for prostate cancer. *Materials and Method*. Cross-platform gene expression data of prostate cancer cohorts were collected from both The Cancer Genome Atlas (TCGA) and Gene Expression Omnibus (GEO). Based on the expression of tyrosine metabolism-related enzymes (TMREs), an unsupervised consensus clustering method was used to classify prostate cancer patients into different molecular subtypes. We employed the least absolute shrinkage and selection operator (LASSO) Cox regression analysis to evaluate prognostic characteristics based on TMREs to obtain a prognostic effect. The nomogram model was established and used to synthesize molecular subtypes, prognostic characteristics, and clinicopathological features. Kaplan–Meier plots and logrank analysis were used to clarify survival differences between subtypes.

**Results:**

Based on the hierarchical clustering method and the expression profiles of TMREs, prostate cancer samples were assigned into two subgroups (S1, subgroup 1; S2, subgroup 2), and the Kaplan–Meier plot and logrank analysis showed distinct survival outcomes between S1 and S2 subgroups. We further established a four-gene-based prognostic signature, and both in-group testing dataset and out-group testing dataset indicated the robustness of this model. By combining the four gene-based signatures and clinicopathological features, the nomogram model achieved better survival outcomes than any single classifier. Interestingly, we found that immune-related pathways were significantly concentrated on S1-upregulated genes, and the abundance of memory B cells, CD4+ resting memory T cells, M0 macrophages, resting dendritic cells, and resting mast cells were significantly different between S1 and S2 subgroups.

**Conclusions:**

Our results indicate the prognostic value of genes related to tyrosine metabolism in prostate cancer and provide inspiration for treatment and prevention strategies.

## 1. Introduction

Prostate cancer is one of the most common cancers in men [1, 2]. About 20–30% of prostate cancer patients will progress to a biochemical recurrence [3], followed by clinical recurrence and metastases, resulting in patient death. Thus, it becomes urgent to subclassify a subgroup of patients as potential responders to adjuvant therapy. Previous studies suggested that prostate-specific antigen (PSA), Gleason score, and tumor stage are critical factors in predicting the recurrence of prostate cancer patients [4]. However, the values of these features were limited by intratumor heterogeneity, sampling error, interobserver variability, or subjective evaluation. Several studies focused on gene expression profiles to generate predictive signatures to identify patients with different risks and obtain moderate outcomes [5–7]. Nevertheless, a few disadvantages limit the usage of these signatures, i.e., several signatures comprise many genes (e.g., 157- or 30-gene signatures), resulting in technical challenges and overload during clinical use. Moreover, few of these signatures were validated by more than two independent cohorts.

Recently, accumulating evidence suggested that tumor cells will generate more oxidative stress and increase ROS production than normal cells [8]. Regulation of cell signaling and metabolism could be controlled by redox homeostasis and is finely tuned in cancer cells [9, 10]. Like other amino acids, tyrosine is the basis of proteins and serves as an alternative energy source for cellular activity. Besides, tyrosine metabolism and dysregulation participate in the progression of diverse diseases, such as Huntington's disease [11], esophageal cancer [12, 13], and hepatocellular carcinoma [14]. Some sporadic studies revealed that tyrosine metabolism pathway-related genes participate in prostate cancer progression and serve as prognostic markers for cancer patients [15–20].

Here, we dissect the potential prognostic value of tyrosine metabolism-related genes in prostate cancer by integrating several large-scale datasets. These findings will shed light on the genomic variations, clinical relevance, and potential effects of tyrosine metabolic enzymes on prostate cancer progression.

## 2. Materials and Method

### 2.1. Data Collection

TCGA level-3 RNA-seq gene expression data, somatic mutation data, and the corresponding clinical pathology and survival information of patients with prostate cancer were downloaded from UCSC Xena (https://xenabrowser.net/datapages/). Gene expression array, clinical pathology, and survival information of GSE116918 were downloaded from Gene Expression Omnibus (GEO) database (https://www.ncbi.nlm.nih.gov/geo/). The gene expression profile and clinical pathology and survival information of the Memorial Sloan Kettering Cancer Center (MSKCC) were downloaded from the following website: https://www.mskcc.org/.

### 2.2. Enzyme Gene Selection

The genes (42 genes) belonging to the tyrosine metabolic pathway were extracted from the Kyoto Encyclopedia of Genes and Genome (KEGG) database. Among them, 19 genes are annotated as enzymes, referring to the MetaCyc database (https://metacyc.org/).

### 2.3. Bioinformatics Analysis

Unsupervised hierarchical clustering analysis of prostate cancer samples was performed using the *R* package “ConsensusClusterPlus v1.42.0” [21]. Genes whose adjusted *P* value was less than 0.05 and |log2Foldchange| higher than 1 were identified as differentially expressed genes (DEGs), which were obtained using the *R* package “DESeq2 v1.18.1.” Gene Set Enrichment Analysis (GSEA) was applied by “clusterprofiler v3.6.0” package [22], and the hallmark gene sets were downloaded from the GSEA MSigDB (https://www.gsea-msigdb.org/). A protein interaction network was constructed using the webserver GeneMANIA (https://genemania.org/). Immune cell decomposing analysis was executed using the CIBERSORT algorithm [23]. Somatic mutation data of TCGA prostate cancer was transformed into the format of “maf” and exhibited using the “maftools v2.2.20” *R* package. Cox regression analysis and Kaplan–Meier estimation were achieved by *R* package “survival c3.1–7.” The nomogram was produced by *R* package “rms v2.4.1.” The LASSO Cox regression algorithm was used for prognostic model construction by applying *R* package “glmnet v2.0–18” with default parameters. In this process, the regression coefficients were determined by the value of *λ* that gives the minimum mean partial likelihood deviance.

### 2.4. Statistics

R software (version 3.4.3) was adopted to execute all bioinformatic analyses. The Kaplan–Meier (KM) curve was used to display the difference of overall survival, and the logrank test was used to determine its significance. The receiver operating characteristic curve (ROC) and the area under the ROC curve (AUC) were used to estimate the discriminative power of the classification system, and a decision curve analysis was used to evaluate the usability of the nomogram. The chi-squared test was performed to identify genes which were differentially mutated between the subgroups.

## 3. Results

### 3.1. Expression of Tyrosine Metabolism-Related Enzymes (TMREs) in Prostate Cancer and Normal Tissue

**Figure 1:**
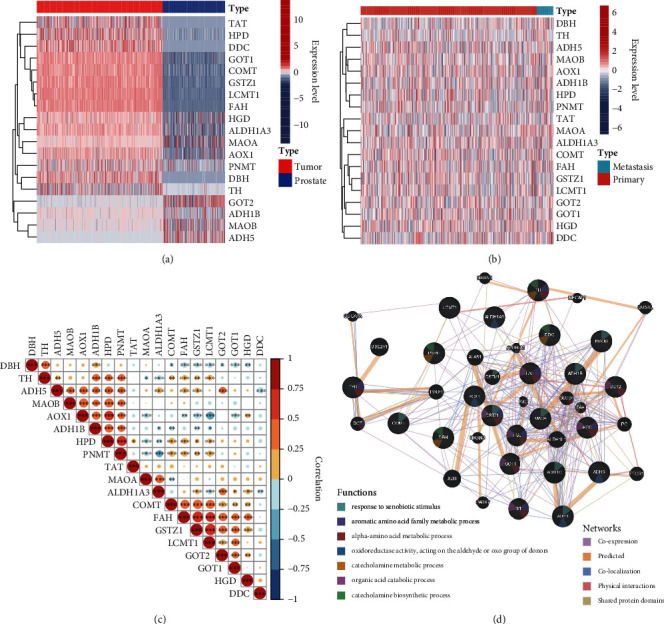
Expression of tyrosine metabolism pathway-related genes in prostate cancer. (a) Heatmap showed the expression landscape of tyrosine metabolism-related genes between prostate cancer and normal prostatic tissues; (b) heatmap showed the expression landscape of tyrosine metabolism-related genes between metastatic prostate cancer and primary prostate cancer tissues; (c) Pearson analyses showed the correlation between genes encompassed in tyrosine metabolism pathways; (d) the regulation network and pathway enrichment of tyrosine metabolism pathway-related genes using GeneMANIA.

**Figure 2:**
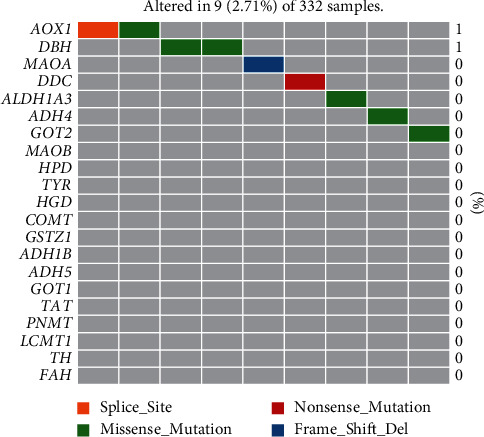
Mutational landscape of genes in the tyrosine metabolism pathway in prostate cancer.

Firstly, we downloaded the TCGA prostate cancer expression data from UCSC Xena (https://xenabrowser.net/datapages/) and performed DEG analysis. Most of TMREs, including *TAT*, *HPD*, *DDC*, *GOT1*, *COMT*, *GSTZ1*, *LCMT1,* and *FAH* were significantly upregulated (*P* < 0.05), while *GOT2* and *ADH5* were significantly deregulated in prostate cancer tissues (Figure 1(a)). When comparing the tumor samples in which patients were found to have tumor metastases with other tumor samples, we found that no enzymes were differentially expressed in these two groups (Figure 1(b)). The co-expression analysis suggested that majority of the enzymes were positively correlated. Particularly, HPD was significantly related to most TMREs (13 out of 18 genes) (Figure 1(c)). We further constructed the regulation network and performed pathway enrichment analysis on TMREs-related genes based on gene expression, sublocations, as well as protein-protein interactions (Figure 1(d); see Methods). Genes from this regulation network were enriched in many metabolic-related pathways, including “aromatic amino acid family metabolic process,” “alpha-amino acid metabolic process,” “catecholamine metabolic process,” “organic acid catabolic process,” “catecholamine biosynthetic process,” and other pathways including “response to xenobiotic stimulus” and “oxidoreductase activity, acting on the aldehyde or oxo group of donors.” We also investigated mutations in these TMREs and found that 2.71% of the samples of TCGA prostate cancer have nonsilent mutations (Figure 2).

### 3.2. Prognostic Subtyping of Prostate Cancer Based on the Expression of TMREs

**Figure 3:**
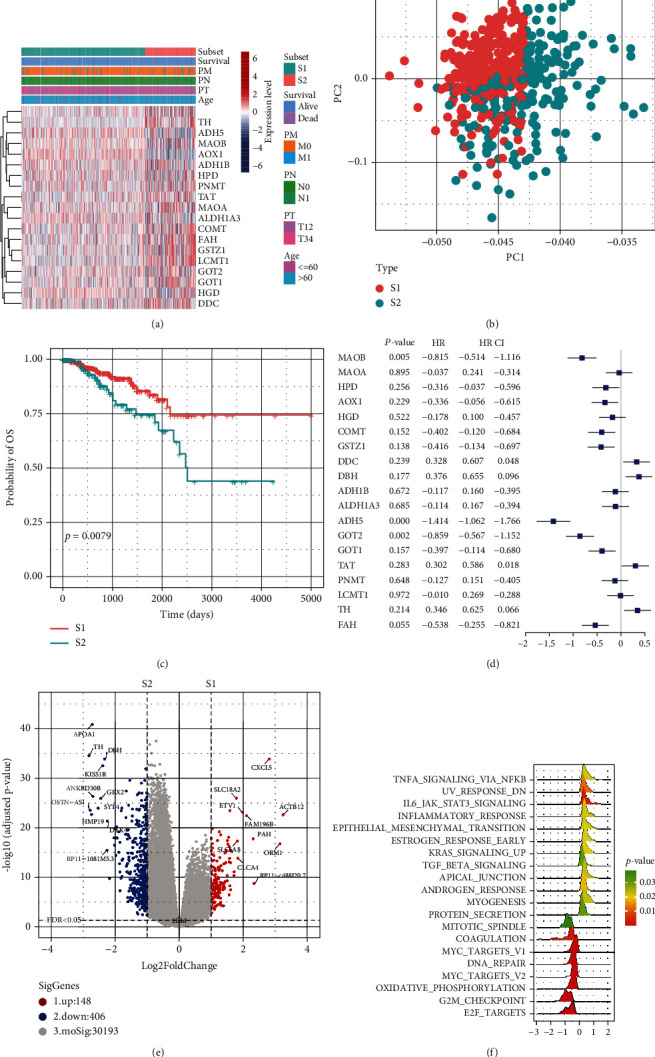
Establishment of the molecular subgroup of prostate cancer. (a) The unsupervised hierarchical clustering method established the molecular subgroup of prostate cancer based on the expression of 19 tyrosine metabolism-related enzymes; (b) principal component analysis (PCA) showed the clustering of two molecular subgroups; (c) Kaplan–Meier plot and logrank analysis showed the survival difference between S1 and S2 subgroups; (d) univariate Cox regression analysis revealed the prognostic role of tyrosine metabolism-related genes in prostate cancer; (e) the differentially expressed genes between S2 and S1 subgroups; (f) GSEA revealed the difference between S2 and S1 subgroups at the pathway level.

Based on the expression of 19 TMREs, we applied an unsupervised hierarchical clustering method to establish the molecular subgroups of prostate cancer. As a result, 498 prostate cancer samples were classified into two subtypes, S1 (354 samples) and S2 (144 samples) (Figure 3(a)). The prostate cancer subtype S1 was characterized with high expression of AOX1, while S2 samples expressed high expression of *MAOB*, *DBH*, *DDC*, *PNMT*, *HPD*, *TH*, *ADH1B*, *ADH4,* and *TAT*. Compared with S2 (144 samples), we found the subtype S2 showed a higher level of regional lymph node involvement (*X*-square = 3.0881, *P*-value = 0.07). PCA analysis further shows that there are apparent differences in expression between the two subtypes (Figure 3(b)). Notably, the patients in the S2 group had worse overall survival (OS) outcomes than those in the S1 group (Figure 3(c)). We also examined the prognostic value of TMREs, and multivariate cox regression analysis results showed that *MAOB*, *ADH5*, and *GOT2* were independent favorable prognostic factors (Figure 3(d)).

**Figure 4:**
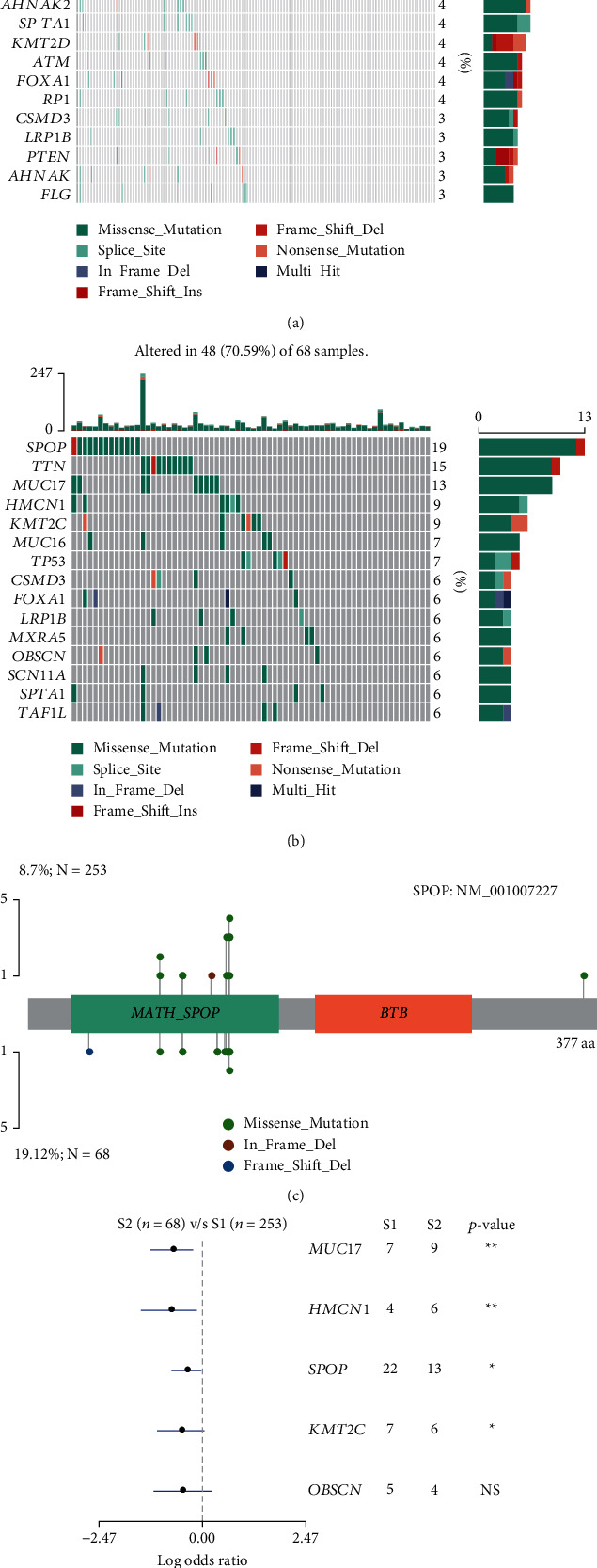
The frequently mutated genes between S1 and S2 subgroups. (a) Frequently mutated genes in the S1 group of TCGA prostate cancer. (b) Frequently mutated genes in the S2 group of TCGA prostate cancer. (c) The forest plot shows the differentially mutated genes between S1 and S2. (d) Mutations of SPOP in S1 (up) and S2 (down) groups, respectively.

We identified 554 DEGs between the S2 and S1 subgroups, including 148 upregulated genes and 406 downregulated genes (Figure 3(e) and Supplemental Table 1). Hallmark GSEA shows that hallmark gene sets including TNFA signaling via NFKB, UV response, DN, epithelial-mesenchymal transition, estrogen response, early KRAS signaling, TGF beta signaling, apical junction, androgen response, and more were upregulated in S1 whereas cell division related pathways such as mitotic spindle and G2M checkpoint were significantly upregulated in S2 (Figure 3(f) and Supplemental Table 2). Particularly, we noticed that immune-related terms including IL6, JAK, STAT3 signaling, and inflammatory response were also highly expressed in S1. We also compared the S1 and S2 samples at the genomics level. Of the fifteen most prevalent mutated genes in the two subsets, eight genes were shared (Figures 4(a) and 4(b)). The *TTN* gene is too long and was excluded in the mutation frequency analysis in most of the other studies. After removing it, *SPOP* was the most frequently mutated gene in both S1 and S2. 5 genes, including *MUC17*, *HMCN1*, *SPOP*, *KMT2C*, and *OBSCN,* were mutated with a significantly higher frequency in the poor prognostic subgroup (S2) (Figures 4(c) and 4(d); *P* < 0.05, chi-squared test).

### 3.3. A Four-Gene-Based Signature Showing Robustness Risk Stratification of Prostate Cancer

**Figure 5:**
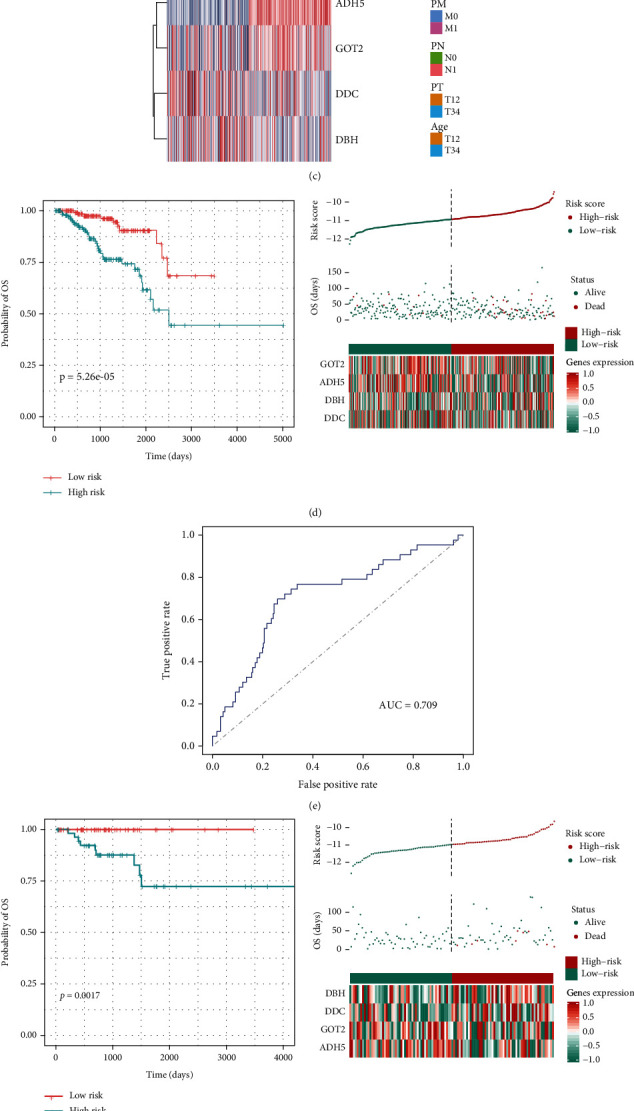
Establishment of the signature based on tyrosine metabolism-related genes. (a) Partial likelihood deviance of different numbers of variables; (b) the LASSO coefficient profiles of the selected four features; (c) the heatmap plot showed the distribution of four selected genes between different clinicopathological features; (d) the Kaplan–Meier plot and log-rank analysis showed the survival outcome difference between low- and high-risk subgroups in the TCGA training cohort; (e) the ROC curve showed the predictive value of the four gene-based signatures in the training cohort; (f) the Kaplan-Meier plot and logrank analysis showed the survival outcome difference between low- and high-risk subgroups in the TCGA validation cohort; (g) the ROC curve showed the predictive value of the four gene-based signatures in the validation cohort.

**Figure 6:**
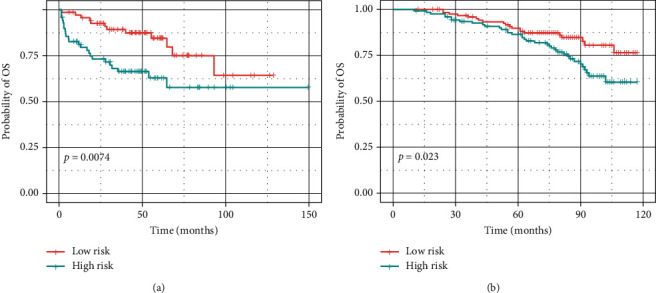
External validation of the established four tyrosine metabolism-related genes in MSKCC (a) and GSE116918 (b) cohorts.

To expand the application of TMREs in the prognosis of prostate cancer, we built a prognostic model to predict the risk score of each patient. We randomly divide the TCGA sample into a training set and a test set at a ratio of 3 : 1. All 19 TMREs were used to build predictive models by using the LASSO Cox regression algorithm. 1,000-time alteration and cross-validation were applied in this process, and as a result, a four-gene signature including *ADH5*, *DBH*, *DDC,* and *GOT2* was adopted (Figures 5(a)–5(c)). The prognostic model is displayed as follows: Risk_score_=−0.763 × Exp_ADH5_+0.053 × Exp_DHB_+0.013 × Exp_DDC_ − 0.148 × Exp_GOT2_. The patients were divided into two subgroups based on the median risk score, which showed significant differences in survival outcomes (*P*=5.26 × 10^−5^) (Figures 5(d) and 5(e)). The ROC curve further proved the robust prognostic prediction performance of our model (AUC = 0.709) (Figure 5(f)). We next divided the test data into high-risk and low-risk groups, and the results also showed significant prognostic differences (*P*=1.75 × 10^−3^) (Figures 5(g) and 5) and robustness (AUC = 0.806) (Figure 5(g)). We further applied this model to two external validation datasets (MSKCC dataset and GSE116918), including 140 and 109 prostate cancer samples, respectively. The high- and low-risk groups also present significantly different OS times in both datasets (*P*=7.35 × 10^−3^ and *P*=0.02, respectively) (Figure 6).

**Figure 7:**
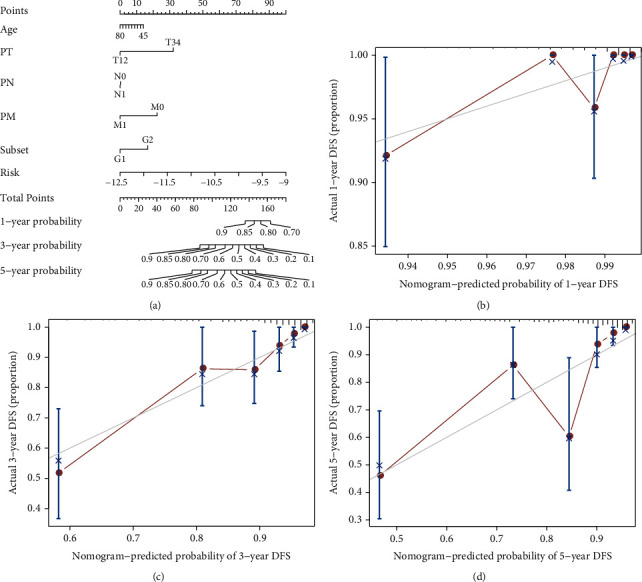
Nomogram model establishment. (a) The establishment of a nomogram model by integrating prostate cancer patients' age, pathological grade, *T*, *N*, *M* stages, and a risk score of four-gene models. An unsupervised hierarchical clustering method established the molecular subgroup; (b–d) the calibration curve for 1-year, 3-year, and 5-year PFS from the prognostic nomogram model.

We evaluated the predictive power of the signature for different clinical prostatic cancer subtypes. A new nomogram model was constructed to quantitatively predict the probability of OS in the TCGA training dataset referring to the multivariate Cox regression analysis (Figure 7(a)). Besides, we assessed the 1-, 3-, and 5-year OS proportions for prostate cancer patients, and a calibration analysis was executed to validate our findings through resampling (Figures 7(b)–7(d)). These results prove that the nomogram model has an application in predicting the prognosis of prostate cancer.

### 3.4. Association between Risk Score and Tumor-Infiltrated Immune Cells

**Figure 8:**
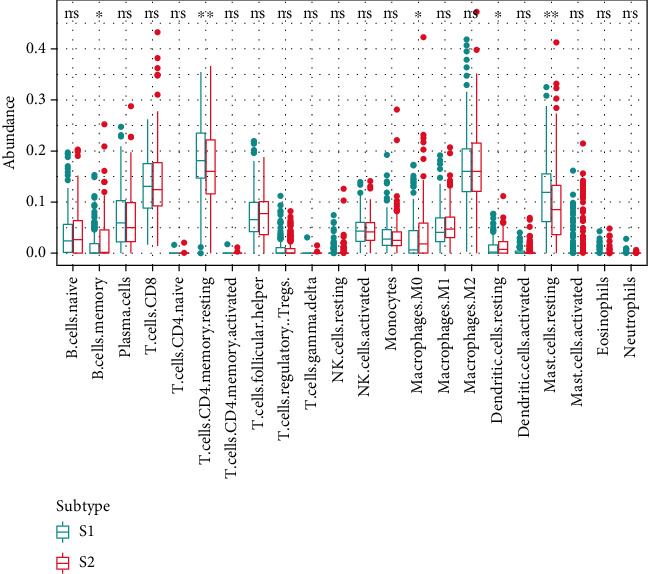
A boxplot presented compositional differences of 22 immunocytes between the low-risk group (S1) and the high-risk group (S2).

**Figure 9:**
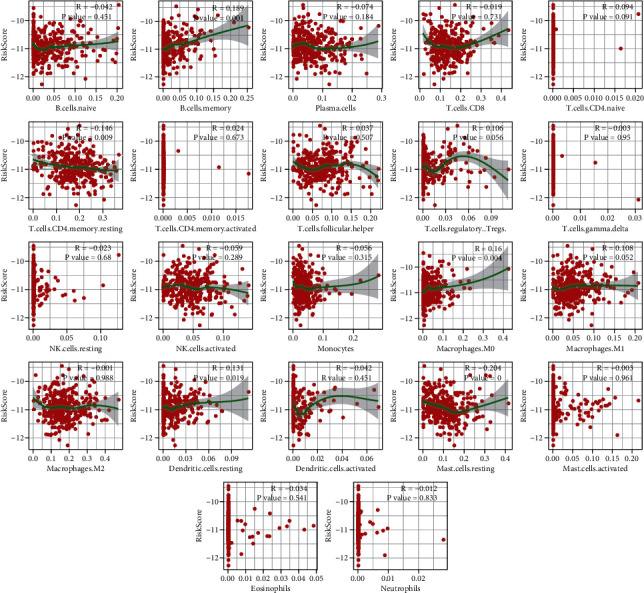
The correlation between the risk score derived from the nomogram model and ratios of infiltrated immune cells.

Considering the immune-related pathways enriched in the GSEA above, we performed infiltrated immune cell decomposition for each TCGA prostate cancer sample using CIBERSORT. We found that the abundance of memory B cells, CD4+ resting memory T cells, M0 macrophages, resting dendritic cells, and resting mast cells were significantly different between S1 and S2 subgroups (Figure 8) and were significantly associated with the risk scores (Figure 9).

## 4. Discussion

We retrieved available public prostate cancer datasets to explore the prognostic role of tyrosine metabolism-related genes in prostate cancer. Our exploration indicated that most tyrosine metabolism-related enzymes, including *TAT*, *HPD*, *DDC*, *GOT1*, *COMT*, *GSTZ1*, *LCMT1,* and *FAH*, were significantly upregulated, while GOT2 and ADH5 were significantly deregulated in prostate cancer samples. To study the effect of tyrosine metabolism on the progression of prostate cancer, we used an unsupervised consensus clustering method to establish the molecular subgroups of prostate cancer based on the expression of 19 tyrosine metabolism-related enzymes. S1 and S2 subtypes of prostate cancer patients showed different survival outcomes. We further conducted a GSEA and found that the differences between the two groups mainly focused on TNF-*α* signaling through NF-*κ*B, UV-response DN, and EMT pathways. In addition, at the genomic mutation level, significant differences were also observed between the two groups. We used LASSO Cox regression analysis and established features based on four genes to obtain a better prognostic effect. Both the Kaplan–Meier and ROC analyses proved the significance and robustness of the signature in the overall survival prediction in MSKCC, TCGA, and GSE116918 cohorts. Notably, by combining the four-gene-based signature and clinicopathological features, the nomogram model achieved better survival outcomes than any single classifier, as suggested by Kaplan–Meier, ROC, CSS, and other analyses. These results proved the critical role of tyrosine metabolism in prostate cancer.

Tyrosine metabolism is a necessary process that often occurs in various diseases, including cancer and chronic diseases [24]. Patients with tyrosinemia type I have an increased risk of developing liver cancer [24], a result consistent with Nguyen et al.'s [25] work that the tyrosine catabolic enzymes' expression is associated with the prognosis of liver cancer patients, and the upstream regulators might participate in the reprogramming of tyrosine catabolism and drive the initiation of liver cancer. Consistently, tyrosine is decreased in serum samples derived from esophageal cancer patients compared with the healthy control [26, 27]. Here, we revealed that the aberrated expression of tyrosine metabolism-related genes in prostate cancer served as prognostic markers for prostate cancer patients. Of note, *ADH5*, *DBH*, *DDC,* and *GOT2* were selected to establish the prognostic signature. The staining of ADH5 might be useful in classifying the subtypes of breast cancer. Besides, Pontel et al. [28] reported that endogenous formaldehyde could be cleared by ADH5, and Adh5 (-/-) mice, thus, the formaldehyde adducts were accumulated in DNA, which will contribute to the initiation of fatal malignancies. DDC plays a vital role in the enzymatic synthesis of dopamine, and dysregulation of this gene has been reported in various malignancies. Gilbert et al. [29] considered that the expression of DDC could be used to precisely discriminate neuroblastoma from other small round cell tumors among children. Consistently, Avgeris et al.'s [30] study confirmed that DDC expression could distinguish prostate cancer from normal tissues. GOT2 is a transaminase that plays an important role in the malate-aspartic acid shuttle and the aspartic acid that generates nucleotide biosynthesis. Yang et al. [31] found that suppressing the function of GOT2 will result in a profound induction of senescence, thus decreasing pancreatic cancer growth. The GOT2 mutation carriers are more susceptible to paragangliomas and pheochromocytomas, which are uncommon neuroendocrine tumors described as a strong genetic determinism [32]. Previous studies also showed that genes from tyrosine metabolism reprogramming (TAT, HPD, HGD, GSTZ1, and FAH) may be used as prognostic biomarkers in liver cancer [25]. However, this study did not construct a robust prognostic model, which limits its clinical application. Interestingly, although these five genes did not appear in our prognostic model, three of them (TAT, HPD, and FAH) were significantly highly expressed in prostate cancer, showing a high degree of concordance. This result suggests that the tyrosine metabolism reprogramming pathway may play a similar role in the progression of different tumours. Another interesting study shows that the activation of tyrosine aminotransferases in the tyrosine metabolic pathway affects treatment resistance in the glioblastoma core [33], highlighting the deleterious role of genes in this pathway in tumour therapy. Therefore, a more in-depth study of these genes is necessary and of great interest. In the future, we will investigate the functions of these key genes in tumour progression at the cellular level and im animal models, and the biological mechanisms behind them.

We also investigated the mutational difference between S2 and S1 subgroups. We found that MUC17, HMCN1, SPOP, and KMT2C were significant and frequently mutated in the S2 than in the S1 subgroups. MUC17 is commonly mutated in diverse cancers [34]. A total of 63% of pancreatic ductal adenocarcinoma carried mucin gene alteration events, and the frequency of MUC17 is 15%. Besides, for these, patients with global upregulation of MUC1/4/16/17/20/21 commonly have a poor prognosis. Silencing the expression of MUC17 significantly increases the chemotherapy sensitivity in breast cancer [35]. In addition, survival analysis showed that deregulation of MUC17 was significantly associated with poor prognosis after chemotherapy. HMCN1 encodes an extracellular protein [36]. Lee et al. [37] identified that the frameshift mutations of HMCN1 will lead to a premature stop of amino acid synthesis and result in the loss of function of this gene; besides, they also indicated that HMCN1 is inactivated in gastric cancer and colorectal cancer with mutations. Kikutake et al. [38] found that the intratumor heterogeneity of the HMCN1 expression is correlated with the prognostic value of breast cancer patients. SPOP seems to play pleiotropic tumorigenic effects since many pathways have been altered upon its mutations [39–41]. SPOP is the substrate-binding subunit of E3 ubiquitin ligase. For normal cells, the protein degradation process is essential. Thus, that is why proteasome pathways are prevalently mutated in diverse cancers. Nowadays, mutations or variations of SPOP are one of the potential causes of the dysfunction of the proteasome pathway in diverse cancers [42–44]. Finally, either downregulation of SPOP or gene mutations will promote the stabilization of downstream proteins of SPOP that will subsequently promote the progression of cancers [45]. As one of the critical epigenetic regulators, KMT2C frequently mutates in diverse cancers and is a crucial biomarker in detecting the occurrence or progression of diverse cancers [46, 47]. Besides, KMT2C is regarded as a tumor suppressor, and deletion of this gene is correlated with unfavorable prognosis of breast cancer [48], acute myeloid leukemia [49], and gastric adenocarcinoma [50]. Functional studies are required to verify the function of these highly mutated genes in prostate cancer.

## 5. Conclusion

To sum up, we successfully established a nomogram model based on tyrosine metabolism-related genes and validated the feasibility of this nomogram in independent prostate cancer cohorts. This prognostic model would be helpful for clinicians to classify patients at different risks and thus develop appropriate treatment strategies.

## Data Availability

The data used to support the findings of this study are available from the corresponding author (1931320211@stmail.ntu.edu.cn) upon request.

## Abbreviations

TMREs: Tyrosine metabolism-related enzymes
LASSO: Least absolute shrinkage and selection operator
PSA: Prostate-specific antigen
TCGA: The Cancer Genome Atlas
GEO: Gene Expression Omnibus
MSKCC: Memorial Sloan Kettering Cancer Center
KEGG: Kyoto Encyclopedia of Genes and Genome
DEGs: Differentially expressed genes
GSEA: Gene set enrichment analysis
KM: Kaplan–Meier
ROC: Receiver operating characteristic curve
AUC: Area under the ROC curve
OS: Overall survival.

## Supplementary Materials

Supplementary Materials Supplemental Table 1: top 100 differentially expressed genes between S1 and S2. Supplemental Table 2: significant hallmark gene set enrichment analysis results between S1 and S2.
