# Supplementary material for: Gene Expression Analysis Reveals Prognostic Biomarkers of the Tyrosine Metabolism Reprogramming Pathway for Prostate Cancer
Source: J Oncol. 2022 Jul 6;2022:5504173. doi: 10.1155/2022/5504173 (PMC9279037; doi:10.1155/2022/5504173)
Supplement: Supplementary Materials — Supplemental Table 1: top 100 differentially expressed genes between S1 and S2. Supplemental Table 2: significant hallmark gene set enrichment analysis results between S1 and S2. [file 5504173.f1.zip › 5504173.f1/Supplemental Table 1.pdf]

supplemental Table 1. Top100 Differencially expressed genes between S1 an

Gene _symbol	BaseMean	Log2FoldChange	LfcSE	Stat
TSPAN6	3408.461655	0.029438623	0.051573841	0.570805328
TNMD	12.77626702	0.177506155	0.213832327	0.830118427
DPM1	1379.971508	-0.067329167	0.028090227	-2.396889425
SCYL3	1003.670781	0.123456864	0.043750402	2.821845231
C1orf112	204.8614888	-0.105589668	0.044517769	-2.371854408
FGR	269.0020519	0.168763694	0.082530176	2.044872578
CFH	1127.365461	0.330783294	0.099643777	3.31965833
FUCA2	3452.154	-0.077983974	0.04574866	-1.704617682
GCLC	2595.098894	0.134625827	0.049977261	2.69374162
NFYA	1904.498181	-0.072626101	0.045305169	-1.60304229
STPG1	670.375862	-0.036391928	0.04544593	-0.800774201
NIPAL3	7636.92732	0.401916667	0.099358578	4.045112918
LAS1L	2631.438153	-0.339158665	0.030652791	-11.06452794
ENPP4	2948.44379	0.057070762	0.072986695	0.781933769
SEMA3F	3193.707249	0.027074888	0.075561675	0.358315083
CFTR	541.9143115	1.515282757	0.170793854	8.871998125
ANKIB1	2391.736499	0.033639022	0.056940437	0.590775611
CYP51A1	295.0651608	0.24878242	0.085728809	2.901969846
KRIT1	1375.760332	0.065209529	0.046446094	1.403983037
RAD52	417.4174643	0.056241382	0.046402992	1.212020589
MYH16	9.410934193	-0.116432262	0.104942332	-1.109488041
BAD	3158.042685	-0.657152825	0.057650582	-11.39889319
LAP3	3759.945621	0.022381252	0.041457547	0.539859539
CD99	14964.81967	-0.265242577	0.045118706	-5.878771842
HS3ST1	680.3570199	-0.879142739	0.129775257	-6.774347882
AOC1	1146.5622	0.955086427	0.241731734	3.951017973
WNT16	26.05574606	0.425750578	0.145079069	2.934610631
HECW1	154.1697012	0.734975705	0.160949649	4.566494606
MAD1L1	2077.475732	-0.499943897	0.062411308	-8.01046977
LASP1	5503.594615	0.056362526	0.055906535	1.008156318
SNX11	1211.622952	-0.217109667	0.034224346	-6.34372005
TMEM176A	458.0002048	-0.078610728	0.101428046	-0.775039363
M6PR	2385.092864	0.450005195	0.057087433	7.882736524
KLHL13	353.8729862	0.559824092	0.079211395	7.067469153
CYP26B1	148.3780356	0.061491643	0.130368838	0.471674395
ICA1	4082.619233	-0.151831392	0.046080824	-3.294893163
DBNDD1	1927.820466	-0.442446542	0.075431888	-5.865510666
ALS2	1197.771485	0.114957599	0.038476904	2.987703967
CASP10	368.2165196	0.024048551	0.090889977	0.264589689
CFLAR	5303.532497	0.159976592	0.031033738	5.154925016
TFPI	797.2303099	-0.015059013	0.132374595	-0.1137606
NDUFAF7	519.2541328	-0.143270162	0.031625397	-4.530224905
RBM5	5488.766701	-0.093831351	0.043412714	-2.161379556
MTMR7	46.98051652	0.01493364	0.064621034	0.23109565
SLC7A2	4314.896378	0.097903285	0.07383735	1.325931714
ARF5	8411.887567	-0.472712385	0.048407199	-9.765332333
SARM1	729.1575055	0.168878785	0.04626034	3.650617004
POLDIP2	6877.576466	-0.224661193	0.031636175	-7.101401822
PLXND1	2140.092903	-0.026659616	0.073251993	-0.363943898
AK2	5064.643364	-0.17282562	0.028382571	-6.089146061
CD38	2844.721166	1.175345845	0.182801634	6.4296244

FKBP4	15642.6712	-0.339232853	0.052597954	-6.449544605
KDM1A	5840.787388	-0.091532289	0.033554616	-2.727859807
RBM6	4486.867527	-0.318173419	0.059775125	-5.322839904
CAMKK1	710.1199549	-0.442694595	0.069651156	-6.355882918
RECQL	585.6686866	0.280377426	0.07438706	3.769169368
CCDC132	1181.503719	-0.029699505	0.045300835	-0.655606137
HSPB6	2935.139023	0.34631678	0.129020122	2.684207511
ARHGAP33	579.4789161	-0.417234252	0.076452966	-5.457397867
NDUFAB1	2510.129826	-0.532105546	0.047582644	-11.18276534
PDK4	3877.872773	0.28889551	0.137273626	2.104523051
SLC22A16	24.66358962	-0.038389708	0.182344292	-0.21053419
ZMYND10	198.6054952	-0.542690846	0.158013139	-3.434466594
ABCB5	15.70967323	0.094893611	0.152789102	0.621075784
ARX	297.4206427	-0.693163183	0.174627705	-3.969376928
SLC25A13	1633.193711	-0.114146787	0.050290937	-2.269728768
ST7	1772.117773	-0.0353732	0.045262484	-0.781512555
CDC27	2281.172188	0.068970466	0.035573856	1.938796473
SLC4A1	4.266492365	-0.662997999	0.158521447	-4.182386741
CALCR	2.92007471	0.024379531	0.154164769	0.158139448
HCCS	874.8906035	-0.028208108	0.029652049	-0.951303831
DVL2	2161.254531	0.004439754	0.03377524	0.131449971
PRSS22	776.9200269	0.097850982	0.085870581	1.139516942
UPF1	10120.25917	0.009012232	0.027584039	0.326719096
SKAP2	1790.706832	0.01287367	0.058703516	0.219299819
SLC25A5	10101.54623	-0.155021331	0.049579989	-3.126691541
CCDC109B	229.8413735	-0.048787805	0.078106379	-0.624632777
HOXA11	742.9886209	-0.346245417	0.063416071	-5.459900202
POLR2J	2302.789111	-0.689531377	0.062926369	-10.95774938
DHX33	1273.54706	0.045656839	0.039779247	1.147755209
MEOX1	189.5509663	0.159277442	0.108499839	1.467997025
THSD7A	1218.195847	-0.129917682	0.149822	-0.867146895
LIG3	3410.327877	-0.10300211	0.05293393	-1.945861757
RPAP3	1378.67514	-0.040420621	0.028860253	-1.400563636
ACSM3	2804.026783	0.009747482	0.107170434	0.090953092
AC004381.6	750.6066895	-0.302180359	0.0618405	-4.886447527
CIAPIN1	1905.028368	-0.139718026	0.040628074	-3.43895274
SPPL2B	2829.20372	-0.380146312	0.055218938	-6.88434669
FAM214B	1552.608247	-0.344118826	0.052101005	-6.604840497
COPZ2	641.1585801	-0.372323031	0.094884017	-3.923980475
PRKAR2B	1311.745378	-0.113175473	0.079566852	-1.422394762
MSL3	1242.69121	-0.24151968	0.036235978	-6.665190133
CREBBP	5566.2546	0.209668708	0.044367935	4.725680974
BZRAP1	1856.721092	-0.076295553	0.065845271	-1.158709676
MPO	8.872469504	0.212099413	0.116933475	1.813846826
PON1	104.1752602	-0.873430201	0.286420269	-3.049470643
GCFC2	1640.991276	-0.019722515	0.045488004	-0.433576176
WDR54	743.8431456	-0.45212366	0.059393317	-7.612365836
CROT	2473.603065	0.142849156	0.058657692	2.435301333
ABCB4	*51.20108407*	*-0.023349716*	*0.082118442*	*-0.284341933*
