# Supplementary material for: Gene Expression Analysis Reveals Prognostic Biomarkers of the Tyrosine Metabolism Reprogramming Pathway for Prostate Cancer
Source: J Oncol. 2022 Jul 6;2022:5504173. doi: 10.1155/2022/5504173 (PMC9279037; doi:10.1155/2022/5504173)
Supplement: Supplementary Materials — Supplemental Table 1: top 100 differentially expressed genes between S1 and S2. Supplemental Table 2: significant hallmark gene set enrichment analysis results between S1 and S2. [file 5504173.f1.zip › 5504173.f1/supplemental Table 2.pdf]

**Supplemental Table 2. Significant Hallmark gene set enrichment analysis results between S1 and S2.**

| ID | SetSize | EnrichmentScore | NES | P-value | P-adjust | Q-values | rank |
|---|---|---|---|---|---|---|---|
| G2M_CHECKPOINT | 189 | -0.524814 | -2.017626 | 0.001049 | 0.010028 | 0.006122 | 3584 |
| MYC_TARGETS_V1 | 194 | -0.452682 | -1.741185 | 0.001052 | 0.010028 | 0.006122 | 13717 |
| OXIDATIVE_PHOSPHORYLATIO[illegible] | 184 | -0.468859 | -1.796860 | 0.001053 | 0.010028 | 0.006122 | 10413 |
| E2F_TARGETS | 195 | -0.578985 | -2.226787 | 0.001054 | 0.010028 | 0.006122 | 6864 |
| DNA_REPAIR | 147 | -0.466477 | -1.750368 | 0.001068 | 0.010028 | 0.006122 | 8748 |
| MYC_TARGETS_V2 | 58 | -0.528812 | -1.788342 | 0.001203 | 0.010028 | 0.006122 | 11673 |
